# The Effect of an Educational Intervention on Self-Care in Patients with Venous Leg Ulcers—A Randomized Controlled Trial

**DOI:** 10.3390/ijerph19084657

**Published:** 2022-04-12

**Authors:** Mirna Žulec, Danica Rotar Pavlič, Ana Žulec

**Affiliations:** 1 Department of Nursing, Catholic University of Croatia, Ilica 242, 10000 Zagreb, Croatia; 2 Department of Family Medicine, Medical Faculty, University of Ljubljana, Poljanski Nasip 58, 1000 Ljubljana, Slovenia; danica.rotar@gmail.com; 3 Galenia D.O.O., Čučkova Ulica 17, 1000 Ljubljana, Slovenia; 4 Department of Psychology, Catholic University of Croatia, Ilica 242, 10000 Zagreb, Croatia; azulec@unicath.hr

**Keywords:** patient education, self-care home care, nurse, venous leg ulcer, self-treatment

## Abstract

Background: Although patients with venous leg ulcers are involved in ulcer management, little is known about why and how these patients self-treat their ulcers without direct supervision by health professionals. Yet patients’ knowledge of ulcer management can be important for achieving ulcer closure and/or preventing recurrence. This study thus investigates the effects of an educational intervention on knowledge of self-care among patients with venous leg ulcers, mainly on wound dressing practice, compression therapy, physical activity and nutrition. Methods and participants: This research was conducted in three outpatient hospitals in central Croatia. An educational brochure was made and distributed to patients; patients were surveyed about caring for venous leg ulcers before the brochure was distributed and after 3 months. Results: In total, 208 patients were involved in the study: 112 in the experimental group and 96 in the control group. The educational intervention increased awareness of compression therapy, knowledge of recurrence prevention, appropriate lifestyle habits, and warning signs related to venous leg ulcers. Conclusions: Patient education on illness and self-care is necessary to achieve positive effects in self-care knowledge. In this study, patients learned how to change dressings, learned how to improve their lifestyle, and were empowered to deal with their illness.

## 1. Introduction

A venous leg ulcer (VLU) is the result of chronic venous insufficiency manifesting as an open skin lesion. VLUs usually occur on the medial side of the lower leg between the ankle and the knee [1]. It is estimated that VLUs affect up to 3% of the adult population worldwide [2], with significant financial costs to health care systems [3,4,5]. Besides taking a long time to heal and having a high recurrence rate [6], VLUs can have a significant impact on patients’ quality of life, with personal, social, and psychological effects and broad social and economic impacts [7]. Treatment for VLUs is based on extensive research and is well documented [7,8,9,10].

Like patients with other chronic diseases, VLU patients and their informal caregivers are often involved in self-care in addition to VLU management in the health care setting. Self-care is defined as follows: “Self-care is a deliberate action that individuals, family members and the community should engage in to maintain good health” [11]. Key components of self-care include maintenance, monitoring, and management. Maintenance includes activities patients do to maintain physical and emotional stability. Monitoring refers to the process of observing oneself for changes in signs and symptoms. Management is reflected in patients’ responses to signs and symptoms when they occur [12]. Patients who practice self-care have a better quality of life [13,14], lower hospitalisation rates [15,16,17,18], and lower mortality rates [19]. Although the term self-care refers to a single person, others, such as spouses, relatives, and friends, are often involved in self-care, and their role has been investigated and emphasised in recent research [18,20,21,22,23]. Self care for patients with a VLU should include care for the ulcer itself, application of adequate dressing, application of compression therapy, and correction and adherence to a certain lifestyle involving adequate movement, exercise and nutrition.

Self-care can have multiple benefits for the patient and the health care system, and it is important for all patients, with accent on patients in rural areas or those who are managing diseases in which self-management can significantly influence improvement [24].

Improved knowledge about their illness increases patients’ ability to engage in self-care and helps them avoid unhealthy behaviours. Educated patients become more connected to their health and consequently more adherent to care guidelines and posttreatment care; furthermore, they become more aware of when and why to seek help and how to engage in preventive measures [25,26].

Nowadays, online education is preferred among educators and patients [27,28,29]. However, written educational messages in the form of brochures still have value and can have a positive impact on patients’ knowledge and self-management of disease. Through Pubmed search with “leaflet” AND ”venous leg ulcer” and “brochure” AND “venous leg ulcer” from 2009–2019. We have not found any research on educational brochures on VLUs and their effect on knowledge of self-care. The present research was conducted before the COVID-19 pandemic, which has only highlighted the lack of health care among chronic patients and the ability of these patients to adequately care for their VLUs.

Aim of this study is to investigate the effects of an educational intervention on knowledge of self-care among patients with VLU.

## 2. Materials and Methods

### 2.1. Study Design

An experimental pre–post intervention study was carried out at three hospitals in central Croatia, one university hospital in Zagreb and two general hospitals in the cities of Bjelovar and Koprivnica during 2019. In each hospital, the observational period was 4 months. The study was conducted in such a way that the questionnaire surveyed all participants, only experimental group received the educational brochure, and all respondents were re-examined after 3 months on their scheduled exam.

### 2.2. Participants

All VLU patients who had scheduled an exam at a vascular surgery outpatient clinics were invited to participate in the research. Eligible patients were older than 18 years and able to give informed consent. Patients who could not communicate reliably or who had cognitive impairment, or a history of mental illness were excluded.

We calculated the required sample size for this study using the G*Power program for all statistical analysis, we assumed a type I error of 0.05, and the effect size between 0.3 and 0.5. Recommended sample sizes for this research were *n* = 176 for *t*-test, *n* = 184 for Wilcoxon-Mann-Whitney U test (with each group having 92 participants), and *n* = 210 for one-way ANOVA. Given the very specific population for our sample and the high risk of participants dropping out of the study, we collected a total of 308 participants, from which 208 were included in the final analysis [30].

**Figure 1:**
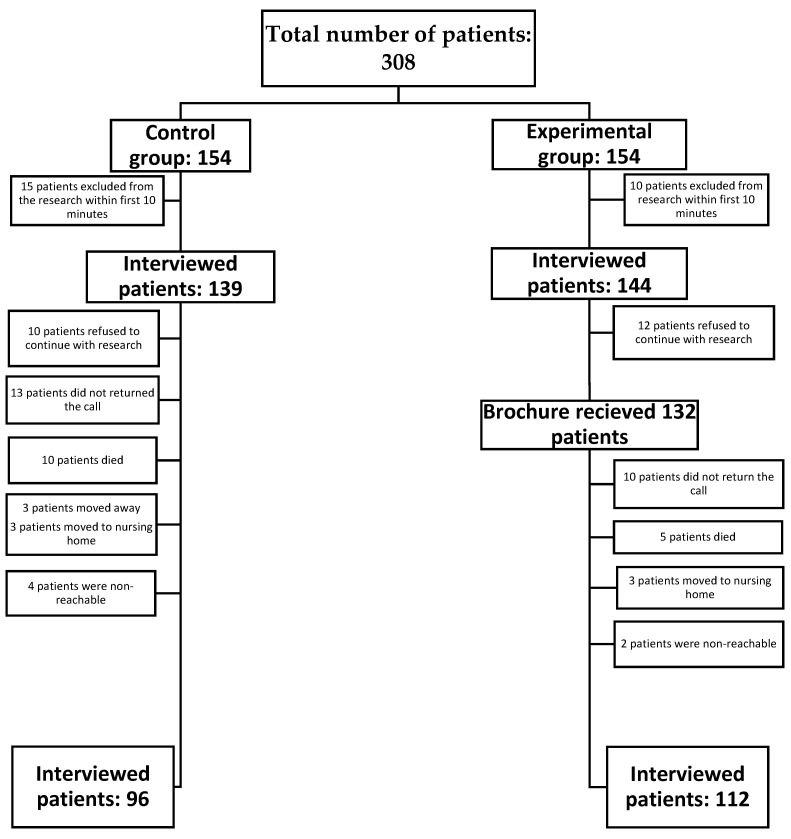
Flowchart of participants and data collection.

Nurses working at the outpatient clinic approached patients after their exam and invited them to participate in the study. Participants were assigned by systematic random sampling, as they were entering exam room one was chosen for the control group, the next for the experimental group, they signed an informed consent form and then were examined to collect data for this first measurement point. The participants in the experimental group received the educational brochure and a short presentation of it. The participants in the control group answered the survey questions and were informed that this would be repeated after 3 months. All participants were informed that they would be examined again after 3 months on their control exam. In total, 308 patients were approached (Figure 1). again after 3 months.

### 2.3. Research Tool

#### 2.3.1. Survey

The survey consisted of standardised and nonstandardised questions that were explained to patients, thus allowing for the opportunity for metacommunication. The questionnaire was prepared using a qualitative study [31] and a literature review. The questionnaire was designed to determine the following information:

- VLU duration and recurrence;
- attitude toward compression therapy and type and frequency of compression therapy used;
- knowledge of wound management and wound care.

The first part of the survey included sociodemographic questions, followed by statements about effectiveness of compression therapy scored on a Likert scale from 1–5.

Multichoice questions were focused on knowledge about VLU care and diet; for questions about necessity for visiting health care provider offered answers were *yes, no, I do not know.*

Last question was open-end type in which participants were asked to answer what they think is the most effective procedure in VLU healing.

#### 2.3.2. Educational Intervention

An educational brochure was created with information on effective self-care of VLUs. It was designed for people with lower literacy levels, as the literature suggests that brochures, especially brochures for wound patients, should not contain medical jargon but should be written at a fifth- or sixth-grade reading level [32]. Written material can be easily distributed, it is inexpensive, and patients like it; a great advantage is that patients can read it at their own pace when they have the time [33].

The brochure contained an introductory section with an explanation of the causes of VLUs and their main characteristics. The central part of the brochure explained wound dressing in a step-by-step manner, with photos of real patients. After that, a section on the types and benefits of compression therapy followed, also with photographs. Special attention was paid to the importance of maintaining regular body and foot hygiene as well as promoting exercise. Descriptions of the the positions of the body at rest and nutrition advice were given. Pictures of leg exercises were shown, and special attention was given to activities for people with limited mobility. The final part included brief tips and tricks (e.g., that compression socks should have folds on their edges and similar points).

#### 2.3.3. Data Analysis

The data collected using the survey questionnaire were statistically processed using the SPSS software package, version 25.0 (IBM Corp., Armonk, NY, USA). Descriptive analyses, bivariate analyses, and multivariate analyses were used. The sample was described using frequencies and percentage distributions for categorical variables. The means and standard deviations were taken for the numerical factors.

The following statistical procedures were used:

- a *z*-test for independent samples
- a *t*-test and a chi-square test to measure the statistical difference between two groups (i.e., self-treating versus not self-treating), effect size d = 0.250
- one-way ANOVA to test differences within one group with more than two variables (i.e., reasons for self-treatment), effect size d = 0.250
- Mann–Whitney test and pairwise comparison to determine the statistical significance of differences between groups, effect size d = 0.498

## 3. Results

In total, 308 patients with VLUs were approached and 208 completed the study. Their descriptive data are presented in Table 1.

Responses to questions about attitudes toward and knowledge of compression therapy, knowledge of procedures for changing dressings, and lifestyle activities were analysed to assess the impact of the educational intervention. One-way analyses of variance and pairwise comparisons (paired *t* tests) were performed. The results of the *t* tests are shown in Table 2 and Table 3.

For all statements in Table 2, the positive responses were considered to be the correct ones. Each correct or positive answer was assigned 1 point, and the points were summed. The same procedure was followed for the second measurement point, and then the results were compared. The details are given in Table 2 and Table 3.

Knowledge improvement was seen in of the following areas:

- compression therapy: the measurement results showed a statistically significant shift, Wilks Lambda = 0.88, F (1.11) = 15.38, *p* < 0.001. There are, therefore, compelling reasons to conclude that the educational brochure influenced the knowledge of compression therapy. In one-way ANOVA, Wilks Lambda = 0.768, F(1.11) = 33.459, *p* < 0.001 participants showed statistically significant increase in awareness that compression therapy is necessary after VLU healing.
- positioning: participants intuitively know that keeping their legs horizontally will lower edema and swelling, so before educational intervention, theiy often that legs should be kept “on the bed”; after education, the answer “on the bed, above the heart level” was more often given, with statistically significant difference. This also include opinion about positive effect of walking on VLU healing.
- hand hygiene: before the intervention, 89% of participants answered that washing hands was obligatory; however, after the intervention all of the answers were correct so in one-way ANOVA Wilks Lambda 0.904, F(1.11) = 11.729, *p* < 0.05 making a statistically significant improvement.
- warning signs (Table 4)
- nutrition (Table 5)
- knowledge of effective VLU treatment (Table 6)

In all claims regarding warning signs of ulcer worsening, participants have acquired improved knowledge to statistically significant degree. Details are presented in Table 3 and Table 4.

Answers regarding effects on nutrition knowledge were analysed separately, results are presented in Table 5.

After the education, the number of participants who responded that they did not know what treatments were effective for VLU, was significantly reduced. A statistically significant shift occurred in the responses *dressings, compression therapy and hygiene*. The details are presented in Table 6.

## 4. Discussion

An intervention study showed that patiens’ knowledge could be improved not only regarding treatment and dressing of the wound, but also with reference to the broader concept of self-care.

An educated patient is a valuable partner in treatment. Currently, the relationship between health care staff and the patient evolved from patient adherence to patient compliance, patients are now expected to be active and to make decisions regarding their own health. Although it sometimes seems that patients with VLUs should be treated by home care nurses, in an aging population and with fewer nurses, is difficult to implement this. Further, there are now improved compression therapy systems that allow the patient or an informal caregiver to self-administer compression to allow the patient to achieve a certain level of independence. In addition to the fact that the patient can travel or continue working with this therapy, the COVID-19 pandemic has indicated the need for patients and informal caregivers to know how to take care of their wounds in certain situations. Currently, it is not a topic for debate that a patient should know how to take care of himself, but then the question arises: which educational interventions are successful?

There is a lack of research on patient education on wound care; recent research is focused on education on acute wounds [34,35,36].

Education on caring for chronic wounds, such as, pressure ulcers [37,38,39], diabetic foot [40,41], or VLU recurrence [42,43], is also focused on prevention. Broader insight into patient education can be found in the research of Weller et al. [44], who assessed the benefits and harms of interventions designed to help people adhere to VLU compression therapy; Clarke Moloney et al. [45], who investigated the brochure as an educational tool; and more recently Protz et al. [46], who studied the effects of education on compression therapy.

*Hand Hygiene and Dressing Change*

Our results showed that good infection prevention knowledge and education help patients to better understand hand washing and wearing gloves, the positive effect of which can be seen in the study of infection of acute wounds [47,48]. This result was surprising for the researchers as the study population was generally elderly and rural; however, one patient clarified this by explaining that hand washing was commonly done before milking cows. That is, in this part of Croatia because, as the population is engaged in cooperating with diary industry which educated them about connection of hand hygiene and satisfactory microbiological purity of milk; they carried this knowledge over to VLU self-care.

As in previous research [31] was found, patients perform wound dressing as see in the hospital or from home care nurses.

*Skin Care*

Skin care also saw a positive change. Possibly because of a lack of adequate dermatological care, patients gave a range of answers in the initial interviews, not knowing when and how to take care of the skin surrounding their VLUs. Qualitative research has shown that patients use different preparations, including homemade remedies such as marigold cream, olive oil, pork fat, and others, and often they do not know the name of the cream they use.

At the end of the second interview, patients were prompted by an open-ended question to indicate what they had learned from the brochure, and skin care was positively affected. Skin care is a significant factor in healing an ulcer and preventing the onset of a new one, so this change is extremely important. Similar finding can be seen in other research [38].

*Physical Activity*

Lifestyle changes are inevitable with chronic illness, and any change in the education of patients with chronic illness is important. Among VLU patients, physical activity is positively associated with increased healing rates [49].

Education correlates positively with physical activity in VLU patients [50].

In our study, educational intervention raised knowledge about benefits of physical activity in VLU healing.

*Nutrition*

Nutrition among patients with chronic wounds is a topic of research in wound care. Research conducted by Barber et al. found that patients with VLUs are at risk for malnutrition, which is also associated with inadequate movement [51]. The typical diet in central Croatia is rich in carbohydrates (in the form of white bread and potatoes); leanness symbolises poverty, and although good nutrition is a symbol of wealth, many patients are overweight but simultaneously malnourished.

In Croatia, 20% are at risk for poverty, and 28.7% of women older than 65 are at risk [52], so adequate nutrition for this group is not just a matter of will but is subject to financial limitations.

The findings of Bobridge et al. on patients with chronic venous insufficiency showed that a change in diet, with skin care and the use of compression therapy, was, after 6 months, the least undertaken activity [53].

This educational intervention showed better nutrition knowledge, similar with research [54].

*Compression Therapy*

Compression therapy proved to be an interesting topic in this study for many reasons. First, many patients used inadequate compression. Although multiple studies have shown that multilayer compression therapy is the most appropriate treatment during the active venous ulcer phase [7,55,56,57], in most cases patients used a long elastic bandage.

This analysis showed that the role of compression therapy became more evident to patients after they read the brochure, and in this population which generally has low health literacy, these brochures proved to be an excellent educational intervention supporting the findings of Weller et al. [46].

*Patient Empowerment*

Patients showed greater knowledge and self-determination, including having better knowledge of warning signs during healing and being more confident changing dressings. We think that is not important just to educate patients, but also to empower them to be self confident in their actions as empowerment develops or strengthens patients’ physical, mental, and social skills which allow them to achieve self-management of their conditions and treatment and to better self-determine their health [58].

VLU patient education is obviously demanding for health care providers [59] it is positive to see effects of this intervention.

*Study Limitation*

The study was conducted at the level of secondary health care. This was done be-cause in Croatia, due to difficulties in obtaining reimbursement for health care, it is necessary for a family doctor to refer a patient for a specialist examination until after 3 months of VLU duration. The specialist doctor then orders a control exam within the following 1–3 months period. Thus, we included all patients referred to outpatient vascular clinic of each hospital. Some patients are certainly never referred to specialists, and it would have been desirable to include all patients with VLUs in our study, but this was not possible because there is no registry of patients with chronic wounds or anything comparable.

Furthermore, it would be desirable for the study to continue for a longer period, such as a full year, and also to measure wound healing over this time.

*Strengths of the Study*

The study had a high participation rate. and a high level of data-completeness. This is the first study on VLU education and can provide starting point for future research.

*Implications for Practice*

It would be helpful to implement standardized VLU education which should be evidence based, but also adjusted to patients’ knowledge and health literacy.

Informal caregivers should also be included in future work of this kind, as they perform a vital part of self-care (for example in applying compression therapy).

## 5. Conclusions

Patient education on illness and self-care is necessary to achieve positive effects in patients’ knowledge. In this study, patients were educated through educational intervention in the form of a brochure, based on their educational needs. Positive effects of education were found on knowledge about compression therapy, warning signs, hand hygiene, skin care, nutrition and physical activity.

## Figures and Tables

**Table 1:** Participant demographic characteristics.

		Study Group	Control Group	*p* Value	*z* Value
		*n* (%)	*n* (%)		
Gender	male	51 (46)	45 (47)	0.037	0.86
	female	61 (54)	51 (53)	0.846	0.14
Age	younger than 65 years	39 (35)	31 (32)	0.148	0.46
	older than 65 years	73 (65)	65 (68)	0.700	1.24
Employment status	employed	10 (9)	10 (10)	0.315	
	retired	88 (79)	76 (79)	0.853	
	not employed	14 (13)	10 (10)		0.68
Housing	lives alone	18 (16)	17 (18)	0.348	0.38
	lives with a spouse	28 (25)	23 (24)	0.479	0.17
	lives with a spouse and children	52 (46)	38 (40)		0.87
	retirement home	5 (5)	3 (3)		0.74
	living with relatives	9 (8)	15 (16)		1.75
Educational level	completed primary school or lower level of education	62 (55)	51 (53)	0.142	0.29
	completed secondary school	44 (39)	39 (41)	0.931	0.28
	completed college or higher level of education	6 (5)	6 (6)		0.31
Residence	urban	55 (49)	48 (50)	0.016	0.14
	rural	57 (51)	48 (50)		0.14

**Table 2:** Comparison of control and experimental groups for each measurement point.

		Control Group		Experimental Group		Wilks’s Lambda	F	*p* Value
Statement	Measurement Point	M	SD	M	SD			
Compression therapy reduces swelling	First	3.24	0.86	3.27	0.96	0.972	(1, 20) = 5.954	<0.05
	Second	3.26	0.87	3.80	1.07			
Compression therapy doesn’t help at my wound	First	2.49	1.20	2.62	1.11	0.972	(1, 20) = 5.954	<0.05
	Second	3.97	1.16	3.07	1.52			
When I’m resting, the best position for legs is …	First	2.28	0.59	2.36	0.68	0.830	(1, 20) = 42.263	<0.001
	Second	2.28	0.59	2.88	0.35			
Before dressing change, I have to wash my hands	First	1.05	0.27	1.15	0.47	0.975	(1, 20) = 5.276	<0.05
	Second	1.02	0.14	1.00	0.00			
When proceed dressing change, it is necessary to use gloves	First	1.40	0.79	1.46	0.72	0.852	(1, 20) = 35.561	<0.001
	Second	1.40	0.79	1.01	0.09			
Number of pair of gloves	First	0.80	0.55	0.71	0.59	0.928	(1, 20) = 15.808	<0.001
	Second	0.84	0.60	1.01	0.09			
When my ulcer heals, I still need to wear compression therapy	First	1.80	0.96	1.81	0.85	0.887	(1, 20) = 26.140	<0.001
	Second	1.78	0.94	1.25	0.53			

**Table 3:** Effects of the educational intervention on experimental group participants.

Statement	Mean	D	SE Mean	Lower	Upper	*t*	df	*p* Value
Compression therapy reduces swelling	−0.53571	1.44527	0.13657	−0.80633	−0.2651	−3.923	111	<0.001
Compression therapy does not help my ulcer	0.33929	1.57431	0.14876	0.04451	0.63406	2.281	111	0.024
The more I walk, the sooner my ulcer will heal	−0.3125	1.64416	0.15536	−0.62035	−0.00465	−2.011	111	0.047
The more I rest, the sooner my ulcer will heal	0.39286	1.6998	0.16062	0.07459	0.71113	2.446	111	0.016
When I’m resting, the best position for my legs is…	−0.52679	0.79367	0.07499	−0.67539	−0.37818	−7.024	111	<0.001
Cream can be applied to the skin around the ulcer	0.09821	1.41396	0.13361	−0.16654	0.36296	0.735	111	0.464
Before changing my dressing, it is necessary to wash my hands	0.15179	0.46904	0.04432	0.06396	0.23961	3.425	111	0.243
Before changing my dressings, it is necessary to disinfect my hands	0.08036	0.72458	0.06847	−0.05531	0.21603	1.174	111	0.243
When my dressing is changed, it is necessary to wear gloves	0.44643	0.73324	0.06928	0.30914	0.58372	6.443	111	<0.001
Even after my ulcer heals, I will still need to wear compression therapy	0.5625	1.02914	0.09724	0.3698	0.7552	5.784	111	<0.001
I need to see a doctor or a nurse when …								
My ulcer smells unpleasant	0.375	0.88149	0.08329	0.20995	0.54005	4.502	111	<0.001
My ulcer is bleeding	0.38393	0.77396	0.07313	0.23901	0.52885	5.25	111	<0.001
My ulcer is leaking a lot	0.45536	0.86876	0.08209	0.29269	0.61802	5.547	111	<0.001
The colour of my ulcer is turning yellow and green	0.51786	0.84876	0.0802	0.35893	0.67678	6.457	111	<0.001
The area around my ulcer is painful	0.48214	0.77089	0.07284	0.3378	0.62648	6.619	111	<0.001
The number of pair of gloves that should be used in a dressing changes	−0.29464	0.59485	0.05621	−0.40602	−0.18326	−5.242	111	<0.001

**Table 4:** Effects of educational intervention on knowledge about warning signs.

		Value	F	Hypothesis DF	Error DF	*p*	Partial Eta Squared	Noncent. Parameter	Observed Powerc
My ulcer smells unpleasant	Wilks′ Lambda	0.846	20.27 0	1	111	0.000	0.154	20.270	0.994
My ulcer is bleeding.		0.801	27.56 0	1.000	111,000	0.000	0.199	27,560	0.999
My ulcer is leaking a lot.		0.783	30.77 0	1.000	111,000	0.000	0.217	30,770	1.000
The color of my ulcer is turning yellow and green.		0.727	41.69 3	1.000	111,000	0.000	0.273	41,693	1.000
the pain is higher		0.717	43.81 1	1.000	111,000	0.000	0.283	43,811	1.000

**Table 5:** Effect of educational intervention on nutrition knowledge.

	%			
	First Measurement	Second Measurement	*z* Value	*p* Value
Meat, fish and eggs	40%	64%	2.56	*p* < 0.050
I dont know	34%	11%	3.86	*p* < 0.050
Bread, pasta and potatoes	18%	24%		
Fruits and vegetables	7%	10%		
All of the above	2%	0%		

**Table 6:** Results of effect on experimental group participants knowledge regarding best VLU treatment.

	%			
	First Measurement	Second Measurement	*z* Value	*p* Value
Dressing change	38%	10%		
I dont know	37%	4%	6.35	*p* < 0.001
Regular check ups	6%	8%		
Resting	4%	1%		
Activity	4%	9%		
Dressing change	4%	55%	9.04	*p* < 0.001
Compression therapy	4%	18%	3.31	*p* < 0.050
Hygiene	3%	26%	5	*p* < 0.001
Nutrition	1%	1%		
Cream	1%			
Desinfictant	1%	2%		
Painless treatment	1%			
Antibiotics		3%		
Ointment		3%		
Gloves		2%		
Medication		2%		
More frequent nurse visits		1%		
Infusion		1%		

## Data Availability

Not applicable.
